# Multilevel Deep Feature Generation Framework for Automated Detection of Retinal Abnormalities Using OCT Images

**DOI:** 10.3390/e23121651

**Published:** 2021-12-08

**Authors:** Prabal Datta Barua, Wai Yee Chan, Sengul Dogan, Mehmet Baygin, Turker Tuncer, Edward J. Ciaccio, Nazrul Islam, Kang Hao Cheong, Zakia Sultana Shahid, U. Rajendra Acharya

**Affiliations:** 1 School of Management & Enterprise, University of Southern Queensland, Toowoomba, QLD 4350, Australia; Prabal.Barua@usq.edu.au; 2 Faculty of Engineering and Information Technology, University of Technology Sydney, Sydney, NSW 2007, Australia; 3 Cogninet Brain Team, Cogninet Australia, Sydney, NSW 2010, Australia; 4 University Malaya Research Imaging Centre, Department of Biomedical Imaging, Faculty of Medicine, University of Malaya, Kuala Lumpur 59100, Malaysia; waiyeec@ummc.edu.my; 5 Department of Digital Forensics Engineering, College of Technology, Firat University, Elazig 23002, Turkey; sdogan@firat.edu.tr (S.D.); turkertuncer@firat.edu.tr (T.T.); 6 Department of Computer Engineering, College of Engineering, Ardahan University, Ardahan 75000, Turkey; mehmetbaygin@ardahan.edu.tr; 7 Department of Medicine, Columbia University Irving Medical Center, New York, NY 10032-3784, USA; ciaccio@columbia.edu; 8 Glaucoma Faculty, Bangladesh Eye Hospital & Institute, Dhaka 1206, Bangladesh; nazrul.islam@hotmail.com; 9 Science, Mathematics and Technology Cluster, Singapore University of Technology and Design, Singapore 487372, Singapore; 10 Department of Ophthalmology, Anwer Khan Modern Medical College, Dhaka 1205, Bangladesh; zshahid28@gmail.com; 11 Department of Electronics and Computer Engineering, Ngee Ann Polytechnic, Singapore 599489, Singapore; 12 Department of Biomedical Engineering, School of Science and Technology, SUSS University, Singapore 129799, Singapore; 13 Department of Biomedical Informatics and Medical Engineering, Asia University, Taichung 41354, Taiwan

**Keywords:** OCT image classification, diabetic macular edema (DME), hybrid deep feature generation, iterative feature selection, digital image processing

## Abstract

Optical coherence tomography (OCT) images coupled with many learning techniques have been developed to diagnose retinal disorders. This work aims to develop a novel framework for extracting deep features from 18 pre-trained convolutional neural networks (CNN) and to attain high performance using OCT images. In this work, we have developed a new framework for automated detection of retinal disorders using transfer learning. This model consists of three phases: deep fused and multilevel feature extraction, using 18 pre-trained networks and tent maximal pooling, feature selection with ReliefF, and classification using the optimized classifier. The novelty of this proposed framework is the feature generation using widely used CNNs and to select the most suitable features for classification. The extracted features using our proposed intelligent feature extractor are fed to iterative ReliefF (IRF) to automatically select the best feature vector. The quadratic support vector machine (QSVM) is utilized as a classifier in this work. We have developed our model using two public OCT image datasets, and they are named database 1 (DB1) and database 2 (DB2). The proposed framework can attain 97.40% and 100% classification accuracies using the two OCT datasets, DB1 and DB2, respectively. These results illustrate the success of our model.

## 1. Introduction

Optical coherence tomography (OCT) is an imaging technique using low coherence light sources to produce high-resolution cross-sectional images of the retina and optic nerve. It is useful to diagnose various pathologies causing optic atrophy or optic nerve swelling [1]. It displays the retinal layers in three dimensions and allows the evaluation of changes in the macula. It is widely used in the detection of diabetic macular edema (DME) and age-related macular degeneration (AMD) [2,3].

DME is an eye disease caused by damage or proliferative blood vessels in the retina, particularly at the macula [4]. DME, which is an important complication of diabetes in the eyes, can lead to irreversible vision loss. On the other hand, AMD is degeneration at the macula region caused by various external risk factors, such as age, genetic variants, family history, smoking, etc. It usually occurs in individuals over the age of 60 years [5]. The prevalence rate of AMD increases with age and is the leading cause of blindness in the elderly [6].

Vision loss is not observed in the early stage of DME and AMD. However, it causes vision loss and blindness in advanced stages [7]. Therefore, early diagnosis of DME and AMD is very important. However, analyzing each section in OCT individually is time consuming and increases the workload of clinicians [8]. Many machine learning techniques have been proposed for the automatic classification and abnormality detection of OCTs [9,10,11]. In these techniques, segmentation of the retinal layer or classification of images with the same characteristics is generally studied [4]. These techniques offer high-performance analysis support at a low cost. Deep learning (DL) methods, which work effectively using large databases, are of immense interest in the literature [12,13,14,15].

In this work, a novel intelligent system is proposed using the most effective DL model. One way to achieve this is to use multiple deep network based feature extractors, and in this study, we present a new hybrid and multileveled deep feature generator. Moreover, we employ hybrid pooling methods to generate compressed images. The utilized pooling methods are maximum, max-min, and max-mean. The routing problem is solved by using these three pooling methods together, and multileveled deep features are generated with three simple pooling functions. The main purpose of the IRF selector is to automatically choose the most appropriate feature vector, and the chosen optimal feature vector is fed as input of the quadratic SVM classifier [16]. As discussed above, our work presents a deep feature engineering framework developed with two widely used public OCT image datasets, and the key contributions of our model are the following:

- An intelligent deep feature generator is presented using transfer learning. Using transfer learning, 1000 features are generated from each pre-trained CNN, 18 pre-trained networks are involved in this framework, and an 18-feature generation function is proposed using these pre-trained CNNs and three maximum pooling methods. The proposed framework generates the best deep features to attain the best classification rates.
- An effective learning model is presented by deploying the proposed multiple CNNs based on a deep feature generator, iterative feature selector (IRF), and classification with SVM. This learning model is developed using two public OCT image datasets. It attained the highest classification performance using both OCT datasets.

The rest of this paper is organized as follows. The literature review is tabulated and discussed in Section 2. The material (datasets) and the proposed DL-based feature engineering method are presented in Section 3. The calculated classification results and performance analysis are given in Section 4. The results and findings are discussed in Section 5. Section 6 ends with the main conclusions of the research and gives an outlook on future directions.

## 2. Literature Review

Deep learning methods are widely used to diagnose many different diseases and have attained high performance [17,18,19,20]. The convolutional neural network (CNN) is one of the most widely used models in deep learning and is often preferred for OCT image analysis [21]. The classification of OCT images has also been conducted using handcrafted methods. Rajagopalan et al. [22] proposed a deep CNN framework with OCT images for the diagnosis and classification of drusen macular degeneration (DMD), DME and normal. The framework achieved a classification accuracy of 95.7%. Alsaih et al. [23] proposed a classification (DME vs. normal) pipeline with spectral domain optical coherence tomography (SD-OCT) images. The pipeline comprised pre-processing, feature extraction, feature representation, and feature classification. With principal component analysis and a linear-support vector machine (SVM), they achieved a sensitivity of 87.5% and a specificity of 87.5%. Sunija et al. [24] proposed a deep CNN method that has six convolutional blocks for the classification of DME, drusen, choroidal neovascularization (CNV) from normal OCT images. The proposed method achieved an accuracy of 99.69%. Das et al. [25] proposed a method that introduces a multi-scale deep feature fusion-based classification approach using CNN for classification of DME and two stages of AMD (drusen and CNV) from healthy OCT images. The proposed method achieved an average sensitivity, specificity, and accuracy of 99.6%, 99.87% and 99.6% respectively. In elsewhere, Lemaitre et al. proposed a method for automatic identification of patients with DME versus normal subjects based on local binary patterns features to describe the texture of OCT images and they compared different local binary pattern feature extraction approaches to compute a single signature for the whole OCT volume [26]. The proposed method achieved a specificity of 75.00% and sensitivity of 87.50%.

Rong et al. [2] proposed a surrogate-assisted classification method to classify retinal OCT images based on CNNs. The proposed method achieved a classification (AMD, DME and normal) accuracy of 100%. Tayal et al. [27] presented an automatic diagnostic tool based on a deep-learning framework (three different CNN models) for the classification of CNV, DME, drusen and normal based on images of OCT scans. The diagnostic tool obtained a classification accuracy of 96.5%.

Srinivasan et al. [28] presented an automated algorithm that utilizes multiscale histograms of oriented gradient descriptors as feature vectors of a support vector machine-based classifier for the detection of retinal diseases via OCT imaging. Their classifier correctly identified 100% with AMD, 100% with DME and 86.67% normal.

Hussain et al. [29] proposed a model with random forest for the detection of DME, AMD and normal, using retinal features from SD-OCT images. The classification method uses features such as the thickness of the retina and the thickness of the individual retinal layers. They obtained an accuracy of more than 96%. The studies described above are summarized in Table 1.

DL models have generally been used to develop automatic classification methods [30,31,32]. The deep models have unique benefits and have achieved good results for computer vision problems. As seen from Table 1, CNNs are the flagship of OCT image classification, but there are various types of CNNs in the literature, and each CNN has its own peculiarity, resulting in variable performance on OCT image datasets. The main goal of hand-crafted methods is to create discriminative features for attaining high classification performance with low time complexity. However, they cannot attain high performance on large/complex datasets. To overcome this problem, deep learning models have been used to classify OCT images. Deep models can attain high performance but there are many deep networks, and each of them has individual performance. The primary purpose of our framework is to use the activity of 18 widely known pre-trained CNNs. In addition, a package learning model is presented without using the trial-and-error method. With this framework, there is no longer the need to propose many methods based on the various CNN models for the classification of OCT images. A general image classification model is now proposed by using these networks together. This model can also be used to solve other computer vision problems, as it is a self-organizing framework. This framework can select the most suitable CNNs based on the context of the problem.

## 3. Material and Method

### 3.1. Material

Two public image datasets (DB1 and DB2) were used to develop the proposed retinal abnormality classification system using OCT images. The details of these databases are given below.

#### 3.1.1. First OCT Image Dataset (DB1)

**Figure 1:**
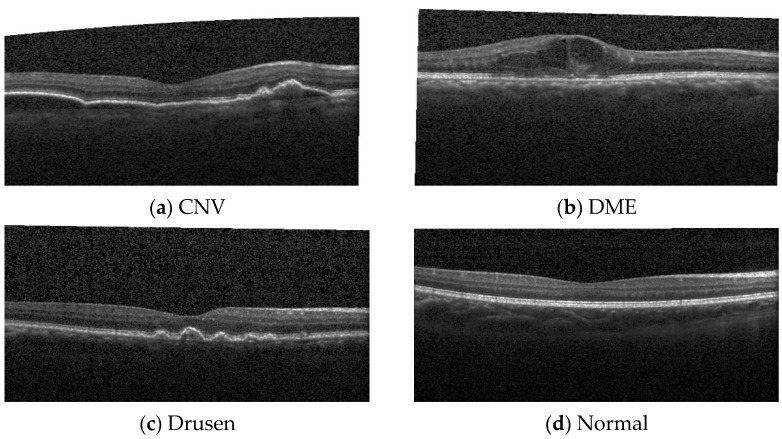
Sample OCT images of the used OCT dataset (*CNV—choroidal neovascularization, DME—diabetic macular edema).* Images reproduced from ref. [33]. (**a**) CNV sample image, (**b**) DME sample image, (**c**) a sample image Drusen class, (**d**) a sample of healthy OCT.

The used OCT image dataset comprised 11,000 OCT images with four classes, named choroidal neovascularization (CNV), diabetic macular edema (DME), Drusen, and normal (https://data.mendeley.com/datasets/rscbjbr9sj/3, accessed on 10 October 2021). There were 2750 OCT images in each of the four categories [33,34]. In this OCT database, 10,000 images out of 11,000 were used for training, and 1000 were used for testing. In the test database, 250 images were used in each category. The dimension of each image was 496 × 1024, and they were stored in jpeg format. Sample images of this dataset are shown in Figure 1.

#### 3.1.2. Second Image Dataset (DB2)

**Figure 2:**
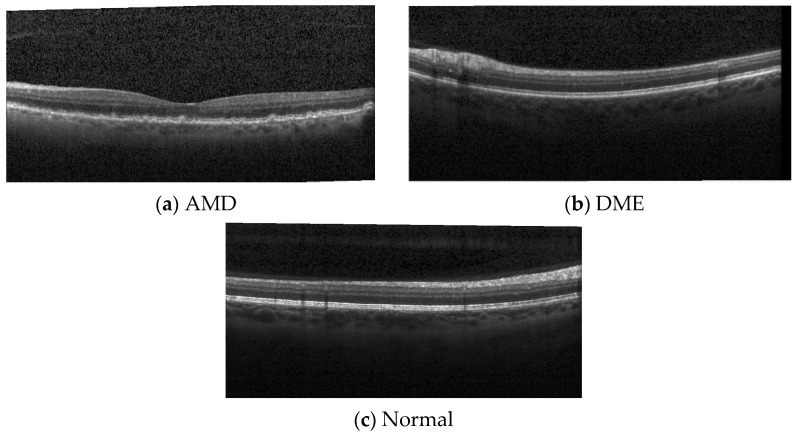
Sample OCT images of DB2 dataset (*AMD—age related macular degeneration; DME—diabetic macular edema).* Images reproduced from ref. [28]. (**a**) AMD disorder, (**b**) DME disorder, (**c**) healthy.

The utilized second database is named DB2, and it contained 3194 images belonging to three categories: age-related macular degeneration (AMD), DME, and healthy classes (https://people.duke.edu/~sf59/Srinivasan_BOE_2014_dataset.htm, accessed on 10 October 2021). It contained 686 AMD, 1101 DME, and 1407 healthy images [28]. The images were stored in tiff format. Sample images of this dataset are depicted in Figure 2.

### 3.2. The Proposed Framework

A new framework is presented to select the best deep features and correctly classify OCT images. The proposed framework consists of three fundamental phases: deep feature extraction using intelligent deep transfer learning, iterative feature choosing, and classification. Pseudocode for this framework is provided in Algorithm 1. The MATLAB (2020b) programming environment was used to implement this algorithm, and 18 pre-trained networks were included, using Get Add-Ons options.
**Algorithm 1:** Pseudocode of proposed frameworkInput: OCT imagesOutput: Results01: Load OCT image dataset02: for *k* = 1 to 1000 do03:    Read each image04:    for *j* = 1 to 18 do//Feature generation using 18 pre-trained networks05:      $X^{j}\left(k,1:1000\right)=CNN^{j}\left(Image\right);$//Extract deep features using *j*th CNN06:      cnt=1000;//Counter defining to calculate the number of features.07:      for *i* = 1 to 3 do//Creating multilevel feature generation network08:         $c^{1}=maxp^{1}\left(Image,\left[3\times 3\right]\right);$//Apply maximum pooling with 3 × 3 sized blocks09:       $c^{2}=maxp^{2}\left(Image,\left[3\times 3\right]\right);$ //Apply max-mean pooling10:       $c^{3}=maxp^{3}\left(Image,\left[3\times 3\right]\right);$//Apply max-min pooling11:               $X^{j}\left(k,cnt+1:cnt+3000\right)=conc\left(CNN^{j}\left(c^{1}\right),CNN^{j}\left(c^{2}\right),CNN^{j}\left(c^{3}\right)\right);$//In Line 11, conc(.) defines concatenation operator and pre-trained CNN generates 3000 features from compressed images.12:       cnt=cn+300013:       $new=maxp^{1}\left(Image,\left[2\times 2\right]\right);$//Compress using images14:       I=new;15:      end for *i*16:    end for *j*17: end for *k*18: for *j* = 1 to 18 do19:    Select the best 1000 features ($f^{j}$) from $X^{j}$ with a length of 10,000.20:    Calculate loss values deploying SVM classifier with 5-fold cross-validation21: end for *j*22: Select the best five features using calculated loss values. We have used quadratic support vector machine (QSVM) as a loss value generator in this phase. An error array with a length of 18 is created using this classifier. The optimal five CNNs are chosen using the created loss array. The minimum loss valued CNNs is the optimal performing CNNs.23: Concatenate these features and obtain 5000 sized feature vector.24: Apply IRF to 5000 sized feature vector for selecting the best feature vector.25: Classify the selected feature vector using SVM and obtain predicted results.

**Figure 3:**
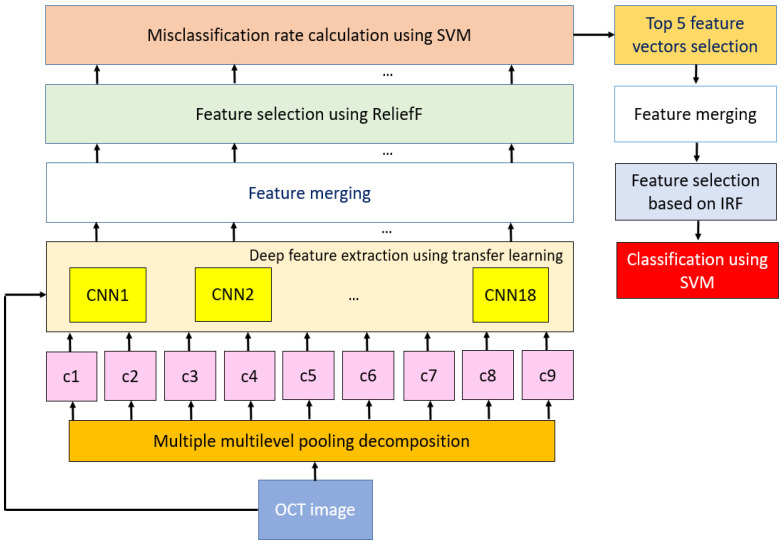
Graphical illustration of the proposed multilevel fused/hybrid deep feature extraction-based OCT image classification model. Maximum, max-min, and max-mean pooling algorithms were used to generate decomposed images (c1, c2, …, c9). By employing transfer learning, 10,000 features were generated from each pre-trained CNN. These networks were trained on the ImageNet dataset. This dataset contained about 1.2 million images belonging to 1000 classes. In this work, we have used the last fully connected layer of each network. Thus, we generated 1000 features for each image. An original and nine compressed images are fed to each pre-trained network. Thus, 10,000 features are generated from an OCT image. One thousand features are selected from the generated 10,000 features utilizing ReliefF, and 18 loss values are calculated in the misclassification rate calculation block. The top five feature vectors were selected using calculated loss values, and the last feature vector with a length of 5000 is determined using the selected feature vectors. The IRF function selected the top features for classification, and results are obtained from SVM with a 10-fold cross-validation strategy. The parameters used in each framework are tabulated in Table 2.

A graphical summary of this framework is presented in Figure 3. More explanations about this framework are provided in Section 3.2.1, Section 3.2.2 and Section 3.2.3.

More details about the suggested model and steps involved in various steps are given below.

#### 3.2.1. Deep Feature Extraction

The most important phase of the proposed framework is deep feature extraction, and this phase is a novel component of the model. For our study, 18 pre-trained commonly used CNNs, three pooling functions, ReliefF [35] and SVM [16] were used together to create the best feature vector. As can be seen from the used methods, the proposed feature extraction model contains machine learning components. This model’s main feature generation functions are pre-trained CNNs; the utilized pre-trained CNNs are listed in Table 3.

In Table 3, the FE layer is the feature extraction layer, the last fully connected layer used for feature generation. In this work, we use the MATLAB programming environment and we use 18 pre-trained networks. Our main aim is to create a general feature generation framework. The results of all pre-trained networks are obtained, and this framework is used to choose the most valuable ones to solve computer vision problems.

The general steps of this generator are summarized below.

***Step 1:*** Read each OCT image.

***Step 2:*** Decompose OCT images using multileveled and multiple pooling-based methods. In our study, maximum, max-min, and max-mean pooling algorithms are utilized as compression methods. The mathematical definitions of this compression method are given below. Furthermore, this compression method is defined in lines 07–15 of Algorithm 1.

(1) $$c^{i}=maxp^{1}\left(I,\left[3\times 3\right]\right),\;i\in \left\{1,4,7\right\}$$

(2) $$c^{i+1}=maxp^{2}\left(I,\left[3\times 3\right]\right)$$

(3) $$c^{i+2}=maxp^{3}\left(I,\left[3\times 3\right]\right)$$

(4) $$I=maxp^{1}\left(I,\left[2\times 2\right]\right),\;i=i+3$$

where $c^{i}$ is the *i*th compressed image, I defines image, and $maxp^{1}\left(.,.\right),maxp^{2}\left(.,.\right),maxp^{3}\left(.,.\right)$ are the maximum, max-mean, and max-min pooling functions, respectively. Moreover, the mathematical definitions of $maxp^{2}$ and $maxp^{3}$ are given below.

(5) t(k)=mean(block(:,k))

(6) $$maxp^{2}\left(block,\left[k\times k\right]\right)=max\left(t\right)$$

(7) i(k)=min(block(:,k))

(8) $$maxp^{3}\left(block,\left[k\times k\right]\right)=max\left(i\right)$$

Herein, t and i are arrays with a length of k, and they store average and minimum values of a matrix (block) with a size of k×k. The mean(.) function is the average value calculation function, and min(.) is the minimum value calculation function. Equations (5) and (6) define the proposed $maxp^{2}$ function, and Equations (7) and (8) explain $maxp^{3}$ function.

**Figure 4:**
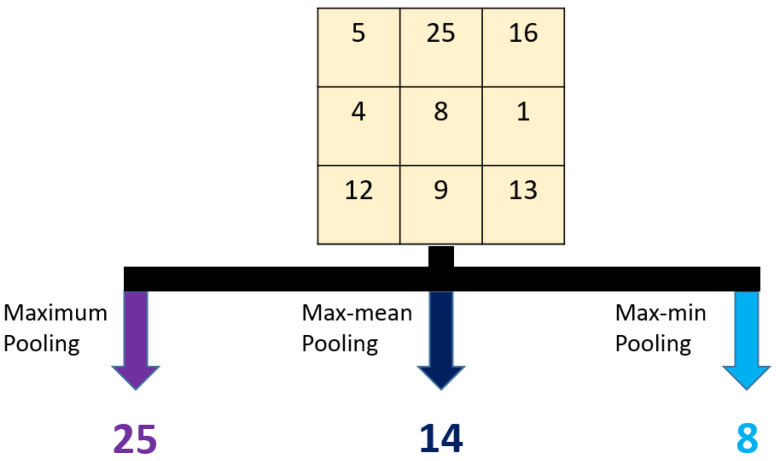
Graphical representation of the used pooling functions with a 3 × 3 dimension sample block. Maximum pooling selects the maximum value. Max-mean and max-min pooling functions select the maximum column, and according to this example, the maximum column is [8,9,25]. By using [8,9,25] vector, max-mean pooling finds $\frac{25+8+9}{3}=14$, and max-min pooling selects 8 as a compressed value.

A graphical representation of the three pooling functions used is depicted in Figure 4.

The general problem of the pooling models is the routing problem [36]. An example of the routing problem caused by pooling is given as follows. Only the peak value can be routed by using maximum pooling. In order to solve this problem using the available pooling methods, our proposed multiple pooling function is presented and by using our proposed pooling function, the average, minimum and maximum values are routed together. The utilized pooling functions take two parameters: input image and used size of the non-overlapping blocks. Nine compressed images are calculated using Equations (1)–(4). This compression model uses three different pooling methods to solve the routing problem, and it has three levels created, using maximum pooling with 2 × 2 sized non-overlapping blocks.

***Step 3:*** Generate deep features from the compressed nine images and original OCT image with 18 pre-trained CNNs as a deep feature generator.

(9) $$X^{j}\left(k,1:1000\right)=CNN^{j}\left(I\right),\;k\in \left\{1,2,\ldots ,dim\right\},j\in \left\{1,2,\ldots ,18\right\}$$

(10) $$X^{j}\left(k,1000\times i+1:1000\times \left(i+1\right)\right)=CNN^{j}\left(c^{i}\right),i\in \left\{1,2,\ldots ,9\right\}$$

where $X^{j}$ is the *j*th feature in the deep feature vector (this extractor generates 18 feature vectors with a length of 10,000), dim defines the number of used OCT images, and $CNN^{j}$ represents *j*th pre-trained deep feature generator.

In Equations (5) and (6), the deep feature generator is defined using 18 pre-trained CNNs (see Table 3). Each pre-trained CNN is employed for the original OCT and compressed images to extract features. Each CNN generates 1000 features, and 10 images are utilized as input for each CNN. Therefore, 10,000 features are extracted in total from an OCT image.

***Step 4:*** Select the most informative 1000 features from the extracted 10,000 features using the ReliefF selector.

(11) $$idx^{j}=RF\left(X^{j},y\right);$$

(12) $$f^{j}\left(k,t\right)=X^{j}\left(k,idx^{j}\left(t\right)\right);t\in \left\{1,2,\ldots ,1000\right\}$$

where $idx^{j}$ is the sorted indexes of the jth feature vector using ReliefF (RF(.,.)) selector, y is the actual output and $f^{j}$ is the selected 1000 features.

***Step 5:*** Calculate the loss value using SVM classifier with 10-fold CV.

(13) $$ℒ\left(j\right)=SVM\left(f^{j},y,10\right)$$

Herein, ℒ is loss value, SVM(.,.,.) defines the SVM classifier and it requires three parameters. These parameters are feature vector, labels, and k value of the used cross-validation method.

***Step 6:*** Select the best five feature vectors using the ℒ vector and merge the selected feature vectors.

(14) $$lf\left(k,1000\times \left(h-1\right)+1:1000\times h\right)=f^{idx\left(h\right)}\left(k,1:1000\right),h\in \left\{1,2,\ldots ,5\right\}$$

where lf is the last feature vector with a length of 5000, and idx is the index of the sorted loss values arranged by ascending terms.

These six steps defined our presented intelligent deep feature generator.

#### 3.2.2. Feature Selection Using Iterative ReliefF

In this phase, an iterative selector is preferred to select the most informative features. The feature selection aims to increase the classification ability and decrease the time complexity of the used classifier. To achieve both of these aims, various feature selectors are presented in the literature. ReliefF [37,38] is a commonly used feature selector, and it is weight based. It generates both negative and positive features. Negative weights are calculated from redundant features. Positive and larger weights are calculated from informative/discriminative features. The indexes of the feature are calculated using ReliefF weights. However, ReliefF cannot select the best feature vector without a trial-and-error method. Therefore, IRF was presented by Tuncer et al. [39] in 2021. IRF can select the best feature vector automatically, and it is a parametric selector. The parameters used are a number of features range and loss generators. These parameters are chosen as [100, 1000] and SVM with 10-fold CV, respectively. IRF selects 794 features from the extracted 5000 features as the length of an optimal feature vector. Steps of the used IRF are given below.

***Step 7:*** Apply ReliefF to generate 5000 features and generate qualified indexes (ind).

(15) ind=RF(lf,y)

***Step 8:*** Select features iteratively by using feature range. The length of the first feature vector is chosen as 100, and the length of the last feature vector is selected as 1000. Therefore, 901 feature vectors are selected.

***Step 9:*** Calculate the loss value of all selected feature vectors using the SVM classifier.

***Step 10:*** Choose the best feature vector.

#### 3.2.3. Classification

In this section, the last phase, classification, is performed using quadratic kernelled SVM with a 10-fold cross-validation strategy. The SVM classifier is one of the widely preferred traditional classifiers and has many kernels [16,40]. The Bayesian optimization technique is used to select the best kernel for this OCT image classification problem. The hyperparameters search range of the Bayesian optimizer is given as follows: multiclass method, One-vs.-One, One-vs.-All; box constrains, 1–1000; kernel scale, 0.001–1000; kernel, Gaussian, quadratic, linear, and cubic; and standardize, false or true. Options of this optimizer are as follows: a maximum number of iterations is 30, and the fitness function is the minimum misclassification ration. The utilized optimizer selects quadratic SVM as the optimum SVM, and this classifier is used in feature extraction, IRF, and classification. The selected hyper-parameters of the used classifier are as follows:

**Kernel:** Quadratic (2nd degree polynomial),
**Kernel scale:** Auto,
**Box constraint:** 1,
**Standardize:** True.

The last step (classification step) is denoted below.

***Step 11:*** Classify the selected features employing quadratic SVM with 10-fold CV (for DB2) or hold-out validation (for DB1).

## 4. Results

The proposed retinal abnormality classification using OCT images based on an intelligent hybrid deep feature generator and IRF was implemented on a simple configured computer. The 18 CNNs were trained on the ImageNet dataset, and each CNN generated 1000 features. This framework was implemented on the MATLAB (2020b) platform. The proposed framework is a parametric framework, and the parameters used are presented in Table 2.

**Figure 5:**
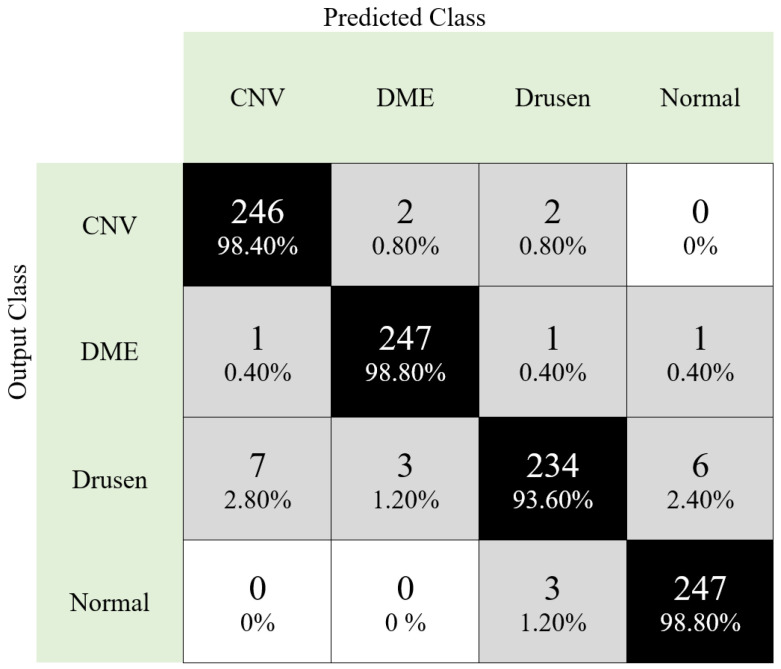
Confusion matrix obtained using our proposed framework for DB1.

In this work, two databases were used to test our proposed transfer learning and the ReliefF-based framework. We included 18 CNNs in this model, and the best five CNNs were selected to create the final features. The performance parameters, namely accuracy (Acc), precision (Pre), Cohen Kappa (CK), F1-score (F1), Matthew coefficient correlation (MCC), and recall (Rec), were used to evaluate the performance of the developed model. The first dataset (DB1) dataset comprises 11,000 images and is a homogenous dataset. The confusion matrix of the proposed classification framework for DB1 is given in Figure 5.

It can be noted from Figure 5 that the best results are obtained for DME and normal classes (our proposal reached 98.80% class-wise accuracy on these classes), and our framework attained 97.40% classification accuracy with this dataset (DB1).

**Figure 6:**
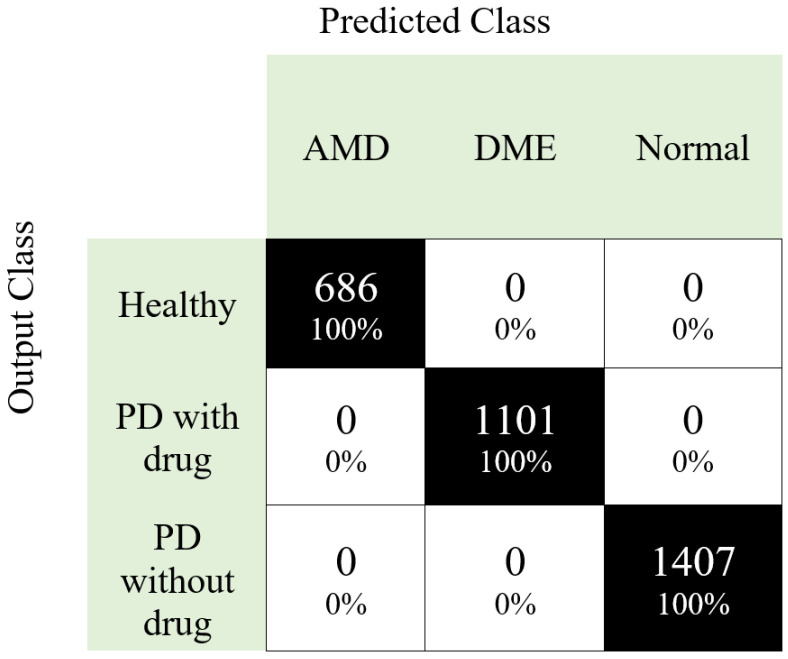
Confusion matrix obtained using our proposed framework for DB2.

The DB2 dataset has 3194 images with three categories, and the results obtained using DB2 dataset are calculated, and the confusion matrix for the DB2 is denoted in Figure 6.

It can be noted from Figure 6 that the proposed framework attained high accuracy (100%) with the DB2 dataset.

The summary of the results obtained using our proposed model with DB1 and DB2 datasets is listed in Table 4.

The time complexity of the proposed framework is calculated using theta (Θ) notation and is given below.

Feature extraction: Θ(p×d×nlogn+p×t)
Feature selection: Θ(i×t+s)
Classification: Θ(t)
Total: Θ(p×d×nlogn+p×t+i×t+s+t)

It can be noted from the above expressions that the proposed model used multiple pooling and pre-trained networks-based deep feature extraction. Herein, p defines the number of the used pre-trained networks, and n is the size of the used images. The used multiple pooling function creates decomposed images, and the decomposed images have lower sizes. Therefore, the complexity is equal to Θ(nlogn). Furthermore, we used the best feature vector selection process in the feature extraction phase, and the complexity of this step is equal to p×t, where t defines the complexity of the used loss value (classifier). d is the time complexity of the used pre-trained network. An iterative feature selector was used. Herein, i is the number of iterations, and s is the time complexity of the feature selector. These results demonstrate that our proposed framework has linear time complexity.

## 5. Discussion

**Figure 7:**
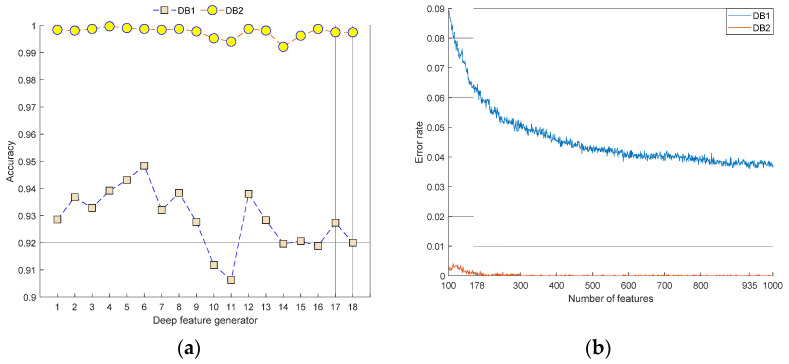
Accuracies and iterative feature selection. (**a**) Classification accuracies obtained using various deep pre-trained CNNs and (**b**) iterative feature selection process using IRF.

This work proposes a new framework for retinal disorder detection using two OCT image datasets (DB1 and DB2) consisting of 11,000 (4 classes) and 3194 (3 classes) images, respectively. The proposed framework is comprised of an intelligent deep feature generator using 18 pre-trained CNNs and multilevel multiple pooling decomposition, IRF selector, and classification. The proposed intelligent feature extractor utilized 18 pre-trained networks as the feature generator. The graph of accuracy rates obtained using 18 pre-trained CNNs is denoted in Figure 7a, and the iterative feature selection process is shown in Figure 7b.

It can be noted from the results that the proposed framework selected deep features of 6th (DarkNet53), 5th (MobileNetV2), 4th (DarkNet19), 8th (EfficientNet b0), and 12th (DenseNet201) CNNs for DB1. DarkNet53 attained 94.84% classification accuracy with DB1. The selected CNNs for DB2 are 4th (DarkNet19), 5th (MobileNetV2), 3rd (ResNet101), 6th (DarkNet53), and 8th (EfficientNet b0) deep feature generators.

It can be noted from Figure 7a that we achieved an accuracy of 90.63% to 94.50% for DB1 and 99.22% to 99.97% for DB2 using QSVM. By merging five deep features, a feature vector of length 5000 is created for classification. These results denote that DarkNet19, DarkNet53, and MobileNetV2 are the top deep feature generators for both OCT datasets. Thereafter, IRF is applied to the feature vector. The lengths of the selected optimal feature vectors are 935 and 178 for the two OCT datasets, respectively.

Figure 7b shows the error rates obtained for various features with two datasets. These graphs clearly indicate the error rate via the number of features. The error rates become 0 for DB2; hence, the classification accuracy is 100%. The error rates are low for DB1, and hence, the accuracy is high.

**Figure 8:**
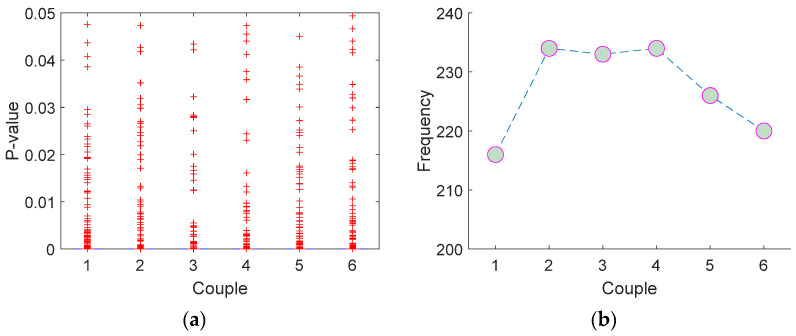
*t*-test results: (**a**) boxplot of the calculated *p*-values, (**b**) number of observations with *p*-values are smaller than 0.05 via couple.

Student’s *t*-test is applied to the generated and selected 935 features of DB1 to validate these classification results. There are four categories in this dataset. Therefore, $C\left(\begin{array}{c} 4\\ 2 \end{array}\right)=6$ couples were used to calculate *p*-values, and obtained *p*-values are shown in Figure 8.

Figure 8 shows the statistical properties of our features, and it validates the classification accuracies calculated using a conventional classifier (QSVM). According to Figure 7b, there are 250 observations in each class, and the number of observations with p-values smaller than 0.05 is calculated as 216, 234, 233, 234, 226, and 220 for 1, 2, 3, 4, 5, and 6 respectively. On the other hand, our framework attained 100% accuracy on the DB2, and the minimum p-values of all couples are calculated as 0.

Furthermore, comparison results are listed in Table 5 to denote the high classification success of the proposed framework.

Table 5 shows that the proposed framework achieved the highest retinal disorder classification performance using both OCT image datasets. We used a maximum number of images and yet achieved optimal performance for both datasets. The model used transfer learning models; hence, the time burden is reasonable.

The benefits of the proposed framework are given below.

- A cognitive transfer learning-based image classification framework is presented.
- An intelligent feature generator is described using 18 pre-trained CNNs and novel multilevel and multiple pooling-based compression methods. Moreover, this feature generator is designed as a learning model. Therefore, it has the best feature vector selection ability.
- The proposed framework is a simple and parametric classification model. It can be extended using more feature extractors, other classifiers, and feature selectors.
- A general computer vision framework is presented with a ten-fold cross-validation strategy. Hence, our developed model is accurate and robust.
- This framework is an extendable framework. By using other effective methods, new-generation image classification methods can be proposed.
- This framework is a fast-learning model since the used CNNs are used in the feedforward mode to extract the features.
- Two OCT image datasets are employed to verify general image classification capability.

## 6. Conclusions

This study has proposed a retinal disorder detection framework using transfer learning, in addition to multilevel multiple pooling decomposition, IRF, and tuned SVM with Bayesian optimization. The main aim of the proposed framework is to select the best pre-trained CNNs to solve the classification problem. We used two public OCT image datasets to evaluate the accuracy and robustness of our developed model. In the proposed framework, DarkNet53, MobileNetV2, DarkNet19, Efficient-Net b0, and DenseNet201 CNNs are selected as the top five deep feature generators for DB1. The selected top five CNNs for DB2 are DarkNet19, MobileNetV2, ResNet101, DarkNet53, and EfficientNet b0. The generated features from these networks are merged, and the best feature vector (the most valuable features) is selected using IRF. We obtained an accuracy of 97.40% and 100% with DB1 and DB2 datasets, respectively. Our proposed framework can potentially be used to detect early stages of retinal disorders. By adopting this framework, screening for retinal disorder in an appropriate patient cohort can be conducted more effectively and enable early treatment. Ultimately, we hope that with this work, irreversible vision loss can be prevented by early diagnosis and prompt medical intervention. This framework can select the most suitable CNNs based on the context of the problem, and new generation CNNs can be included in this framework as part of future work.

## Figures and Tables

**Table 1:** Summary of recent studies involving OCT images.

Study	Method	Purpose	Results (%)
Rajagopalan et al. [22]	CNN	Detecting DMD, DME and normal using OCT images	Acc: 95.70
Alsaih et al. [23]	Local binary patterns and histograms of oriented gradients	Classification of DME and normal using SD-OCT images	Spe: 87.50 Sen: 87.50
Sunija et al. [24]	CNN	Classification of CNV, DME, Drusen and normal using OCT images	Acc: 99.69
Das et al. [25]	CNN	Classification of DME, Drusen, CNV and normal using OCT images	Acc: 99.60
Lemaitre et al. [26]	Local binary patterns	Identification of patients with DME versus normal subjects with SD-OCT images	Spe: 75.00 Sen: 87.50
Rong et al. [2]	CNN	Classification of AMD, DME and normal using OCT images	Acc: 100.0
Tayal et al. [27]	CNN	Identification of CNV, DME, Drusen and normal using OCT images	Acc: 96.50
Srinivasan et al. [28]	CNN	Classification of normal, AMD and DME with SD-OCT images	Acc: 100.0 AMD 100.0 DME 86.67 normal
Hussain et al. [29]	Random forest technique	Classification of normal, AMD and DME with SD-OCT images	Acc: 97.33 for two classes case (DME and normal) Acc: 95.58 for three classes case (DME, AMD, and normal)

**Table 2:** Phases and parameters used in our proposed method.

Phase	Method	Parameter
Feature extraction	Multiple multilevel pooling decomposition	Number of level: 3 Pooling methods: maximum, max-mean and max-min Number of compressed image: 9
	Deep feature generation and feature merging	18 pre-trained convolutional neural networks are used to extract deep features from fully connected layers of these networks. 18 feature vectors with a length of 10,000 are created
	Feature selection using ReliefF	The top 1000 features of 10,000 features generated are chosen.
	Loss value calculation	Quadratic SVM
	Top feature vectors selection	The top five feature vectors have been selected.
Feature selection	Iterative ReliefF	Range of iteration: [100, 1000] Loss value generator: Quadratic SVM
Classification	SVM	Kernel function: Polynomial Polynomial order: 2 Kernel scale: Auto Box constraint: 1 Standardize: True

**Table 3:** Deep CNNs used for deep feature generation.

No.	CNN	FE Layer	No.	CNN	FE Layer
1	ResNet18	fc1000	10	NasNetMobile	predictions
2	ResNet50	fc1000	11	NasNetLarge	predictions
3	ResNet101	fc1000	12	DenseNet201	fc1000
4	DarkNet19	avg1	13	InceptionV3	predictions
5	MobileNetV2	Logits	14	InceptionResNetV2	predictions
6	DarkNet53	conv53	15	GoogLeNet	loss3-classifier
7	Xception	predictions	16	AlexNet	fc8
8	EfficientNet b0	MatMul	17	VGG16	fc8
9	ShuffleNet	node_202	18	VGG19	fc8

**Table 4:** Summary of results obtained using two datasets.

Overall Result	DB1	DB2
Accuracy	97.40	100
Precision	97.40	100
Cohen Kappa	96.40	100
F1-score	97.40	100
MCC	96.53	100
Recall	96.53	100

**Table 5:** Summary of state-of-the-art retinal disorder classification models developed using OCT images.

Study	Method	Classifier	Dataset	Split Ratio	Number of Class	The Results (%)
Rong et al. [2]	Convolution neural network	Convolution neural network	45 subjects 195 Test 1 195 Test 2 207 Test 3 267 Test 4 207 Test 5	72:10:18	3	Acc: 100.0 for volume level
Rasti et al. [3]	Multi-Scale Convolutional Neural Network Ensemble	Softmax	Dataset 1 862 DME, 969 AMD 2311 normal Dataset 2 856 DME 711 AMD 1707 normal	5-fold cross validation	3	AUC: 99.80 Rec: 99.36 F1:99.34 for Dataset 1 AUC: 99.9 Rec: 97.78 F1:97.71 for Dataset2
Fang et at. [41]	Lesion-aware convolution neural network	Softmax	500 CNV 500 DME 500 Drusen 500 Normal	10-fold cross validation	4	Acc: 90.10 Sen: 86.80 Pre: 86.20
He et al. [42]	Label smoothing generative adversarial network	Convolution neural network	1. 37.455 CNV 11.598 DME 8866 Drusen 26.565 Normal 1.581 CNV 4.592 DME 1.563 Drusen 1.168 Normal	Leave-p-out cross- validation	1. 4 2. 4	1. Pre: 87.25 Sen: 87.21 Spe: 95.09 F1: 87.11 2. Pre: 68.36 Sen: 66.68 Spe: 86.73 F1: 67.14
Seeböck et al. [43]	Unsupervised deep learning	Random forest	268 AMD (early AMD, late AMD) 115 control	218 AMD 65 control for training 50 AMD 50 control for testing	3	Acc: 81.40
Alqudah [44]	Automated convolutional neural network	Softmax	250 CNV 250 DME 250 Drusen 250 Normal 250 AMD	95.331 training 40.856 validation 1250 testing	5	Acc: 97.10
Huang et al. [45]	Layer guided convolutional neural network	Convolutional neural network	1. 37.455 CNV 11.598 DME 8866 Drusen 26.565 Normal 2. 1.581 CNV 4.592 DME 1.563 Drusen 1.168 Normal	100:1	1. 4 2. 4	1. Acc: 88.40 2. Acc: 89.90
Fang et al. [46]	Iterative fusion convolutional neural network	Convolutional neural network	37.455 CNV 11.598 DME 8866 Drusen 26.565 Normal	10-fold cross validation	4	Acc: 87.30
Saraiva et al. [47]	Convolutional neural network	Convolutional neural network	5.313 CNV 7.491 DME 1.773 Drusen 2.319 Normal	100:1	4	Acc: 94.35
Our method	Convolutional neural networks, iterative ReliefF	Support vector machine	2750 CNV 2750 DME 2750 Drusen 2750 Normal	10,000 train and 1000 test (10:1)	4	Acc: 97.30 Pre: 97.32 F1: 97.30 Rec: 97.30 CK: 96.40 MCC: 96.41
			686 AMD 1101 DME 1407 Healthy	10-fold cross-validation	3	Acc: 100 Pre: 100 F1: 100 Rec: 100 CK: 100 MCC: 100

## Data Availability

No new dataset was generated from this study. We have utilized the following two public datasets in this study: https://data.mendeley.com/datasets/rscbjbr9sj/3 (accessed on 10 October 2021) and https://people.duke.edu/~sf59/Srinivasan_BOE_2014_dataset.htm (accessed on 10 October 2021).
